# Can a Threshold Value Be Used to Classify Chondrichthyan Reproductive Modes: Systematic Review and Validation Using an Oviparous Species

**DOI:** 10.1371/journal.pone.0050196

**Published:** 2012-12-27

**Authors:** Holly A. Frazer, Megan Ellis, Charlie Huveneers

**Affiliations:** 1 School of Biological Sciences, Flinders University, Adelaide, South Australia, Australia; 2 Safety, Environment and Risk Department, Gladstone Ports Corporation, Gladstone, Queensland, Australia; 3 Biomedical Resources Unit, Faculty of Health Sciences, University of Kwazulu-Natal, Kwazulu-Natal, South Africa; 4 Threatened Endangered and Protected Species Subprogram, SARDI – Aquatic Sciences, Adelaide, South Australia, Australia; Monash University, Australia

## Abstract

The maternal-embryonic nutritional relationship in chondrichthyans has been poorly explored. Consequently, accurately discerning between their different reproductive modes is difficult; especially lecithotrophy and incipient histotrophy. This present study is the first to assess changes in mass throughout embryonic development of an oviparous chondrichthyan other than *Scyliorhinus canicula. Heterodontus portusjacksoni* egg cases were collected and used to quantify the gain or loss of wet mass, dry mass, water content, inorganic and organic matter from freshly deposited eggs (without macroscopically visible embryos) to near full-term embryos. A loss in organic mass of ∼40% found from this study is approximately double the values previously obtained for *S. canicula.* This raises concerns for the validity of the current threshold value used to discern between lecithotrophic and matrotrophic species. Accordingly, 26 studies published in the primary literature between 1932 and 2012 addressing the maternal-embryonic nutritional relationship in sharks were reviewed. Values for changes in mass reported for over 20 different shark species were synthesised and recalculated, revealing multiple typographical, transcribing, calculation and rounding errors across many papers. These results suggest that the current threshold value of −20% established by previous studies is invalid and should be avoided to ascertain the reproductive mode of aplacental viviparous species.

## Introduction

Chondrichthyans (sharks, rays and skates) demonstrate a diverse range of reproductive strategies which are categorised into two distinct modes: oviparity (egg laying) and viviparity (live bearing) [1], [2]. However, these strategies can be further categorised based on the quantity and method by which nutrients are provided to the developing embryos throughout their development [1] [3]. Lecithotrophy refers to the developmental pattern where the yolk, produced by the maternal liver and sequestered in the external yolk sac (EYS), persists for the entire gestation period; therefore, providing the only source of nutrition to the embryo throughout its development [4]. While this is the case for all oviparous species, lecithotrophic viviparity indeed exists, and is the predominant reproductive mode exhibited by chondrichthyans [1]. In contrast, matrotrophy refers to the developmental pattern where the external yolk sac is supplemented by maternal sources once the initial yolk stores are exhausted; consequently this pattern only occurs in viviparous species. Yolk supplementation can occur through a variety of maternal processes such as uterine secretions (histotrophy), placental transfer (placentatrophy), or through the consumption of unfertilised eggs (ovatrophy) or sibling embryos (adelphotrophy) [1], [5].

Although it is now well-known that viviparous chondrichthyans have a wide variety of pathways for providing nutrients to their developing embryos, the specifics of each aplacental strategy is vague. The majority of maternal-embryonic nutritional studies within this group have focussed more extensively on placental species, with particular interest in the uteroplacental interface (e.g. [6]–[9]). The origin and nature of the transferred matter from the maternal organism to the embryo throughout gestation is still poorly explored in most aplacental chondrichthyans [1], [10]–[12].

Previous studies have concluded that the various reproductive strategies described within aplacental viviparity are difficult to classify into distinct categories. Rather, they are placed on a continuum from nil (lecithotrophy) to almost total (matrotrophy) supplementation of the yolk from maternal sources [1], [10], [13]. This can be attributed to the considerable variation in the quantity and quality of uterine secretions exhibited by individual histotrophic species. Due to the graduation of maternal input, it can be especially difficult to discern between the different reproductive strategies at the lower end of the matrotrophy continuum. This is particularly the case between lecithotrophic and incipient histotrophic species [1].

The gain or loss of wet mass, dry mass, water content, inorganic and organic matter between uterine egg and full-term embryo has been used to establish the maternal-embryonic nutritional relationship of chondrichthyan species. This determines the level of dependency of the embryo on the maternal organism, and therefore can aid in the classification of chondrichthyan reproductive modes (e.g. [12]–[16]. Please note that to ensure correct terminology, we used the term ‘mass’ (amount of matter, kg) instead of ‘weight’ (force experienced due to gravity, N). Previous literature mostly used ‘weight’ but is synonymous to ‘mass’ in this paper. The change in organic mass throughout embryonic development is of most value, providing information on the existence and quantity of additional maternal supplies [15]. Based on this principle, Hamlett *et al.* [1] endeavoured to resolve the issue of reproductive classification, by generating a threshold value to differentiate lecithotrophy from matrotrophy. It was concluded that a decrease in mass throughout embryonic development of less than 20% infers the existence of additional maternal supplies and therefore implies matrotrophy [1]. This value was proposed based on several studies investigating the loss in dry and organic mass throughout the embryonic development of an oviparous species, the small spotted catshark *Scyliorhinus canicula* [14], [17]–[19]. Considering that oviparous species do not gain nutritional supplementation other than from the yolk, the use of an oviparous species is a valid way to constitute a threshold value for distinguishing between lecithotrophy and matrotrophy [12], [13], [16]. However, basing a threshold value on one species is dubious as it assumes that the value obtained is representative of all other oviparous species. This is unreliable as it does not take into account inter-species variation or the considerable variation that exists within species, between individuals, or even within individuals [13]. Accordingly, there is a need for further studies to be conducted on additional oviparous species to investigate the existence of inter and intra-species variation.

Furthermore, a number of errors with published mass change values have been highlighted by Huveneers *et al.* [13]. For example, the summary of mass changes tabulated in Hamlett *et al.* [1] referenced Ranzi [14] when in fact it was taken from Table 8 in Needham [20] and included typographical errors [13]. In addition, Hamlett *et al.* [1] was not clear on whether the proposed threshold value referred to dry or organic mass. This emphasises the need for an additional review of the previous literature, and in various cases the correction of mass change values provided, to further investigate the validity of using a threshold value to identify incipient histotrophic from lecithotrophic species.

The objective of the present study was to quantify the gain or loss of wet mass, dry mass, water content, inorganic and organic matter from freshly deposited eggs (without macroscopically visible embryos) to full-term embryos of the Port Jackson shark *Heterodontus portusjacksoni*. *Heterodontus portusjacksoni* was chosen as it is an oviparous species with a known lecithotrophic reproductive mode, allowing for an inter-species comparison with *Scyliorhinus canicula*. Therefore, testing the hypothesis that the organic mass loss of *H. portusjacksoni* throughout embryonic development would be close to 20% and similar to other oviparous sharks. A review of all studies assessing changes in mass throughout embryonic development was also undertaken to assess the validity of the current threshold value and investigate whether a threshold value can be used to classify aplacental viviparous species into specific reproductive modes. In addition, any previously published errors were corrected to what was deemed most accurate.

## Materials and Methods

### Ethics Statement

This research was conducted under Flinders University Animal Ethics Committee (ethics approval number: E335) and PIRSA exemption number 9902364. All specimens were humanely killed, and all efforts were made to minimize suffering.

### Maternal-Embryonic Nutritional Relationship

Port Jackson egg cases were collected from Horseshoe Reef, Gulf St Vincent (35° 8′ 13.99 S”, 138° 27′ 48.00 E”), over a 9-month period – October 2010 to June 2011. All egg cases were transported from the collection site to the laboratory, where they were stored at −20°C until processing.

The entire embryonic system was removed from each egg case (embryo, EYS and internal yolk sac (IYS)) and separated so that the wet mass could be recorded for each specimen individually. The total mass (*W*_T_) of the embryo represents the eviscerated embryo only, excluding both yolk sacs. The total length (TL) of the eviscerated embryo was recorded to the nearest mm. In some cases, ice formed within the egg case, which caused the EYS to burst creating a mixture of yolk fluid and sea water once thawed. Consequently, a value between 0–4 was assigned to each sample depending on the amount of additional seawater present (Table 1). A linear regression, with 95% confidence intervals, between the EYS wet mass and amount of seawater present was calculated to estimate the expected EYS wet mass without seawater. All samples affected by the additional seawater were randomly re-assigned a value within the confidence intervals obtained from the linear regression.

**Table 1:** Seawater scale values assigned to burst external yolk sacs.

Value	Quantity of additional sea water present
0	0ml
1	∼10ml*
2	∼20ml*
3	∼30ml*
4	∼40ml*

* Whilst these quantities were not measured, they were visually estimated at these approximate values, based on the sample container size (70 ml).

Each embryo, EYS and IYS was dried at 60°C until a constant mass was reached. This was achieved within a period of 21 to 157 days and recorded as the dry mass. Dried specimens were transferred into ceramic crucibles and incinerated in a muffle furnace at increasing temperatures to avoid overspill [13]. They were left for two hours at each intermediate temperature (200°C, 300°C and 400°C) and for 15 hrs at a final temperature of 550°C, leaving only the ash mass. The wet mass, dry mass, water content (wet mass – dry mass), inorganic matter (ash mass) and organic matter (dry mass – ash mass) were calculated for each specimen [14], [15]. All measurements of mass were taken to the nearest 0.01 g.

A graphical method, designed by Guallart and Vicent [15], was used to allow a ponderal comparison between freshly deposited eggs and full-term embryos, taking into account the initial size variability of freshly deposited eggs.

### Systematic Review

**Figure 1:**
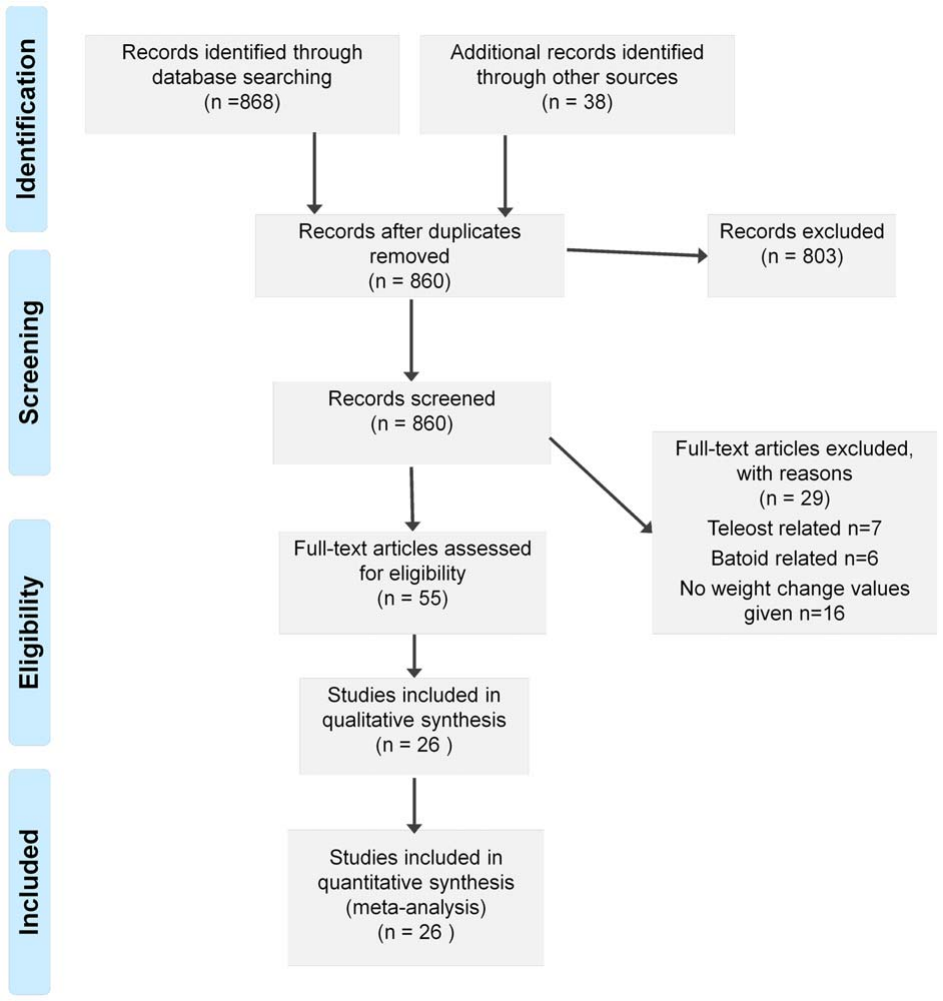
Preferred Reporting Items for Systematic Reviews and Meta-Analyses (PRISMA) flow diagram. Records were identified using ‘Scopus’, ‘Science Direct’ and ‘Web of Knowledge’. Additional records were identified from citations within the papers identified by the database searches. All records were screened and the articles addressing the maternal-embryonic relationship of sharks were assessed for eligibility. Articles reporting mass change values throughout embryonic development of sharks were included in the qualitative synthesis. Articles reporting mass change values for fishes and rays were excluded, along with studies referring to alternative aspects of shark reproductive biology.

A review of the previously published literature was undertaken by searching electronic data base in Web of Science (1932–), SCOPUS (1932–) and Science Direct (1932–). ‘Maternal embryo relationship’ and ‘shark*’ were used as keywords with the most recent searches of all databases undertaken in June 2012. Additionally, any studies cited in the papers identified from the electronic database searches, which mentioned mass change values, were also sourced and incorporated in the review. Studies referring to other aspects of shark reproductive biology, and mass change values for fishes and stingrays were excluded (Fig. 1). All eligibility decisions were made by the first authors. We did not publish a protocol for this review. Mass change values from each study were checked for rounding errors, incorrect calculations, typographical errors, and mis-citations.

All mass change values were examined and recalculated, using ((embryo mass-egg mass)/egg mass)*100, to identify and correct previous errors. When some mass values were absent, they were calculated based on the other reported values (e.g. if dry mass was absent, it was calculated by subtracting water mass from wet mass, or by adding organic mass to inorganic mass). The original and re-calculated mass values were compared to determine whether the original values reported were accurate. In cases where the same author(s) reported different values for a species, the value from the earliest publication was selected, unless additional samples were used in later publications. The information was synthesised and tabulated including species name(s), author(s), whether the IYS was included in embryo mass, whether an average was used or the biological heterogeneity of initial egg size was taken into account, the most accurate mass change values, and the described reproductive mode.

## Results

### Maternal-Embryonic Nutritional Relationship

A total of 82 *Heterodontus portusjacksoni* egg cases were collected and used to quantify the gain or loss of wet mass, dry mass, water content, inorganic, and organic matter throughout embryonic development. The embryos collected ranged in size from 0 mm (not macroscopically visible) to 210 mm. Twenty-nine egg cases (35% of total collected) were freshly deposited and contained only the yolk sac (embryos were not macroscopically visible).. Thirty-nine egg cases (45% of total collected) represented the later developmental stages 13–14 (described by Rodda and Seymour [21]), and were classed as near full-term embryos.

The linear relationship between the EYS wet mass and the additional seawater scale was significant (t-value  = 7.64; df = 24; R^2^ = 0.43; P<0.001). The linear regression produced a 95% confidence interval for EYS wet mass without any seawater between 25.91 g (lower bound) and 45.15 g (upper bound). Consequently, random values within these confidence values were re-assigned to all burst EYS.

**Figure 2:**
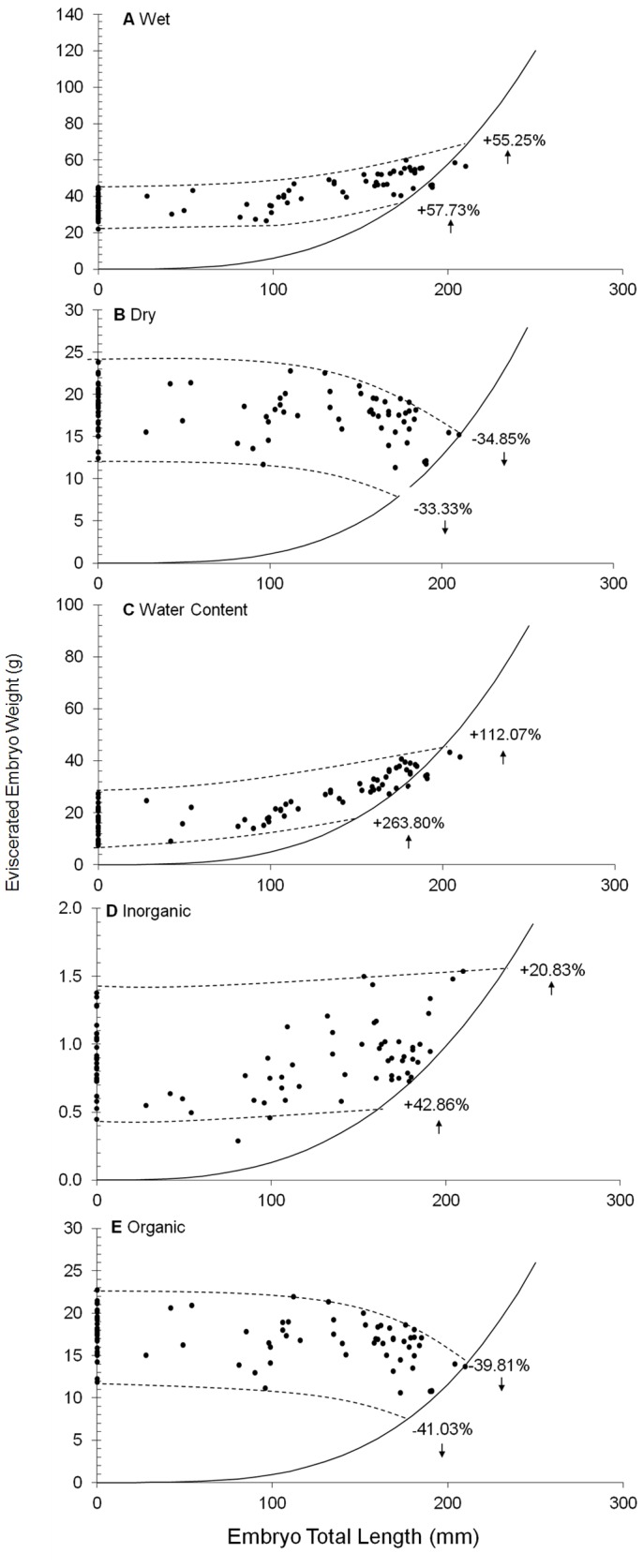
Percentage mass change throughout embryonic development for different constituents. Relationship between embryo total length (TL) and (a) total wet mass (n = 82), (b) total organic matter (*n* = 81), (c) total inorganic matter (*n* = 81) and (d) total water content (*n* = 82) of *Heterodontus portusjacksoni* embryonic system at different developmental stages; the *solid line* represents the eviscerated embryo mass (without external and internal yolk sacs) and embryo TL relationship; the *parallel lines* were designed to include all the data points, following the proposed method by Guallart and Vicent [15] and the intersection of the *parallel lines* with the eviscerated embryo mass–TL relationship indicates the mass change corresponding to the extremes of variability of egg size.

Changes in composition of wet mass, dry mass, water content, inorganic and organic matter are provided for the smallest and largest freshly deposited egg and near full-term embryo (Fig. 2). During the embryonic development of *Heterodontus portusjacksoni*, the total wet mass of the system increased by 57.73% and 55.25%, for smallest and largest extremes, respectively. This was mainly due to the increase of water content (263.80% and 112.07%), and to a minor degree, the slight increase of inorganic matter (42.06% and 20.83%). The total dry mass (inorganic and organic) decreased throughout the embryonic development of *H. portusjacksoni*, showing a loss of 33.33% and 34.85% for the smallest and largest extremes, respectively (Fig. 2). The organic mass decreased more than anticipated for an oviparous species, according to the threshold value proposed by Hamlett *et al.* [1], with a loss of 41.03% and 39.81%, for smallest and largest extremes, respectively (Fig. 2).

The mean percentage change in wet mass, dry mass, water content, inorganic and organic matter was outside the range of values obtained from each respective theoretical TL–embryo body mass relationship (Table 2).

**Table 2:** Comparison of methods.

		Wet	Dry	Water Content	Inorganic	Organic
**Smallest Extreme**	Egg	22.19	12	7.34	0.28	11.87
	Embryo	35	8	26.7	0.4	7
	Change (%)	57.73%	−33.33%	263.80%	42.86%	−41.03%
**Largest Extreme**	Egg	45.09	23.79	27.35	1.44	22.71
	Embryo	70	15.5	58	1.74	13.67
	Change (%)	55.25%	−34.85%	112.07%	20.83%	−39.81%
**Mean**	Egg	34.15±8.90	18.15±4.35	16.00±6.63	0.88±0.29	17.27±4.16
	Embryo	49.79±5.59	17.21±2.65	32.59±5.45	1.00±0.24	16.21±2.64
	Change (%)	45.79%	−5.22%	103.66%	12.65%	−6.13%

Comparison of mean mass values with the smallest and largest mass values obtained when taking into account the biological heterogeneity of egg and embryo size for *Heterodontus portusjacksoni.* Percentage change is over the embryonic development from freshly deposited egg to near full-term embryo.

### Systematic Review

The 868 references obtained were reduced to 55 full text papers assessed for eligibility. Twenty-six of those papers were included in the review, as illustrated in the PRISMA flowchart in Figure 1. The 26 reviewed studies spanned 80 years (1932 to 2012) and changes in mass values produced in the early studies ([14], [20]) are still relied upon in recent publications [1]. When using the electronic data base search, only seven papers were identified as relevant; addressing the maternal-embryonic nutritional relationship in shark species: [11]–[13], [15], [16], [22], [23]. The remainder of the studies reviewed (19) were cited within those seven original papers. Considerable work identified from the database search related to mass change values of teleosts and elasmobranch species other than sharks. Many of the studies identified from the search relating to shark species focussed on other aspects of their reproductive biology, e.g. size at maturity, isotopes comparisons between mother and embryo, and morphological changes in pregnant uteri. These papers were disregarded as this review focussed solely on changes in mass throughout shark embryonic development. Four books were identified in the primary literature and each included a summary of previous mass change values [1], [11], [20], [24]. However, all four books contained at least one major error. Multiple rounding errors, incorrect calculations, typographical errors, and mis-citations were observed in the reported mass change values across many different publications. The majority of key issues observed originated from Needham [20] and Ranzi [14], [25], [26]. A table containing all original and re-calculated mass change values is provided in the supporting information (Table S1).

#### Rounding

Rounding of the original egg and embryo mass values by subsequent authors had a considerable effect on the mass change reported for some species (Table 3). Multiple values recorded in Needham [20] were slightly erroneous due to rounding of the decimal, which was inconsistent and in some cases inaccurate. For the majority of values (14), such rounding only impacted the overall percentage mass change by 0–2% (Table 3). However, it also led to eight overestimations within 2–10% and two overestimations of greater than 20% (Table 3). In the case of the gummy shark *Mustelus antarcticus,* the rounding of embryo dry mass from 2.4 g to 2.5 g by Needham [20], resulted in a 100% overestimation in dry mass gain, due to the minimal difference between egg and embryo mass (Table 4). The embryo dry mass of *Scyliorhinus canicula* was rounded up from 0.538 g in Ranzi [14] to 0.550 g by Needham [20] creating an overestimation of dry mass loss by 20.46%. Needham [20] was not the only author to round values. While he was the only author to round the initial egg and embryo mass, other authors rounded only the final percentage change value. In general, this had less of an effect on the overall percentage change in mass. However, for the spadenose shark *Scoliodon laticaudus*, rounding of the dry mass percentage change from 1,285,614.286%, as calculated in this manuscript, to 1,000,000% as reported by Wourms *et al.* [11], led to a 22.22% underestimation in dry mass change (Table 4).

**Table 3:** Percentage difference caused by rounding.

Species	Wet	Dry	Water Content	Inorganic	Organic
*Scyliorhinus* *canicula*	3.13	20.46	1.29	0.00	1.53
*Dalatias licha*	0.00	0.00	0.00	90.00^a^	0.00
*Centrophorus granulosus*	0.00	0.00	0.00	0.00	0.00
*Squalus vulgaris*	0.00	0.00	0.00	0.00	0.00
*Squalus blainvillei*	0.99	6.67	23.05^b^	78.57^b^	2.91
*Galeorhinus galeus*	0.27	1.03	0.00	1.56	0.00
*Mustelus antarcticus*	0.50	100.00	0.00	7.08	0.00
*Mustelus vulgaris*	0.56	0.39	1.09	5.72	3.76
*Carcharias glaucus*	–	–	–	–	0.14
*Mustelus mustelus*	0.44	3.57	2.50	1.64	1.25
*Mustelus canis* c, d	–	0.69	–	–	–
*Mustelus canis* e	–	0.46	–	–	–
*Eugomphodus taurus* c,d,f	–	1.88	–	–	–
*Eugomphodus taurus* e	–	89.81	–	–	–
*Scoliodon laticaudus* d	–	22.22	–	–	–
*Scoliodon laticaudus* e	–	0.00	–	–	–

a =  level of difference was not due to rounding; reported value was out by a factor of 10.

b =  level of difference was not entirely due to rounding; reported values are possibly miscalculated.

c =  Rounding by Wourms (1981).

d =  Rounding by Wourms *et al.* (1988).

e =  Rounding by Wourms (1993).

f =  Rounding by Stribling *et al.* (1980).

The level of difference rounding has (as a percentage of the recalculated value) on the percentage change reported by Needham (1942), unless stated otherwise, compared to the percentage change values recalculated in the present study based on the original egg and embryo mass values.

**Table 4:** Summary of major errors in previously reported mass changes. Calculated values seen in this table are based on calculations made in this study.

				Reported uterin egg mass	Reported near full- term embryo mass	Percentage Change		Percentage Difference
Species	Study Name	Author	Mass			Reported	Calculated	
*Scyliorhinus canicula*	*Scyllium canicula*	Ranzi 1932	Dry	0.627	0.538		**-14.195**	20.46
		Needham 1942	Dry	0.620 a	0.550 a		**-11.290**	20.46
*Mustelus antarcticus*		Ranzi 1936	Dry	2.300	2.400		**4.348**	100
		Needham 1942	Dry	2.300	2.500 a		**8.696**	100
*Scoliodon laticaudus*		Wourms *et al.* 1988	Dry	0.00007	0.900	1000000 a	**1285614.286**	22.22
*Centroscymnus coelolepis*		Moura *et al.* 2011	Water	47.04	78.09	95.22 b	**66.01**	44.25
*Squalus blainvillei*	*Acanthias blainvillei*	Needham 1942	Water	8.400	17.900	88 b	**113.10**	22.19
*Squalus blainvillei*	*Acanthias blainvillei*	Needham 1942	Inorganic	0.200	1.800	1250 b	**800.00**	56.25
*Mustelus antarcticus*		Needham 1942	Water	2.100	29.800	1480 b	**1319.05**	12.20
*Mustelus antarcticus*		Storrie et al. 2009	Dry	2.267	17.775	784 b	**684.08**	14.61
*Dalatias licha*	Scymnus lichia	Needham 1942	Inorganic	2.000	8.000	30 c	**300.00**	90.00
*Carcharias taurus*	Eugomphodus taurus	Wourms 1993	Dry			120000 c	**1177814.11**	89.81
*Mustelus vulgaris*		Ranzi 1932	Inorganic	0.050	1.450		**2800.000**	94.16
		Ranzi 1936	Inorganic	0.550 c	1.450		**163.636**	94.16
*Mustelus mustelus*	Mustelus laevis	Ranzi 1932	Inorganic	0.069	5.320		**7610.145**	91.18
		Ranzi 1936	Inorganic	0.690 c	5.320		**671.014**	91.18
*Mustelus antarcticus*		Ranzi 1934	Wet	4.400	35.200		**700.000**	9.74
		Ranzi 1936	Wet	4.400	32.200 d		**631.818**	9.74
*Squalus blainvillei*	Acanthias blainvillei	Ranzi 1932	Wet	19.260	38.830		**101.610**	47.32
		Ranzi 1936	Wet	19.260	29.570 d		**53.531**	47.32

a =  Rounding.

c =  Typographical error.

b =  Miscalculation.

d =  Discrepancy.

#### Miscalculations

Three values listed by Needham [20] appear to be a combination of rounding errors and miscalculations. The percentage change in water content and inorganic matter for the longnose spurdog *Squalus blainvillei* were reported by Needham [20] as 88% and 1250%, respectively. However, they were calculated in this study from the original values as 113.095% and 800.000% respectively (Table 4) [26]. Moreover, a similar error is observed pertaining to *Mustelus antarcticus*. The percentage change in water content is reported as 1480% by Needham [20] but recalculated in the present study as 1319.048% (Table 4). Moura *et al.* [12] recorded the water content increase throughout development of the Portuguese dogfish *Centroscymnus coelolepis* for the smallest extreme of variability as 95%. This value was recalculated in the present study, based on Figure 6 in Moura *et al.* [12], as a 66% increase (Table 4).

#### Formula variation

It was also discovered that there is some variation in the way authors calculated the percentage change values. For example, Storrie *et al.* [22] reported the percentage dry mass change for *Mustelus antarcticus* as 784% (Table 4), based on the following formula; (embryo mass/egg mass)*100. It was recalculated in this study as 684.076% (Table 4), using the same formula as the majority of previous studies [12], [13], [15], [16], [26]. The lack of a standard formula being used can account for some of the errors observed across the different studies. In addition, Capapé and colleagues used an alternative method by reporting a chemical balance of development (CBD) rather than a percentage change in organic mass [27]–[29]. This was achieved by dividing the dry mass of term embryos by that of uterine eggs, or oocytes in the case of the angel shark *Squatina squatina* and the smoothback angel shark *Squatina oculata* [27], resulting in incomparable values.

#### Conversion factors

The calculations of the egg and/or embryo dry mass for five different species were based on a conversion factor established by Ranzi [14]. However, the conversion factor varied depending on the study. For example, Stribling et al. [30] and Wourms [22] estimate dry mass based on Ranzi [14], and state that the dry mass of embryos is approximately 30% of the wet mass. Yano [31] referred to the same conversion factor by Ranzi [14], but stated in the methods that the dry mass of embryos is 20% of wet mass (Table S1). Capapé *et al.* [27] also described a standard value of water content, suggesting that oocytes contain 50% water and new borns contain 75% water. Generalisations such as these are not well founded, as pointed out by the variation in water content, inorganic and organic matter among different reproductive modes and genera of chondrichthyans [1]. Considering the inter- and intra-species variability, it is unlikely that the same conversion factor can be used across all species and possibly individuals of varying reproductive modes.

#### Typographical errors

Furthermore, effectual typographical errors are reported throughout the previous literature. The percentage change in inorganic matter or dry mass reported for two species, by two different authors, was out by a factor of 10. Needham [20] recorded the percentage change in inorganic matter for the kitefin shark *Dalatias licha* as a 30% increase, when in fact it was recalculated here to be a 300% increase according to the values of the original study [14]. Wourms [24] reported the percentage change in dry mass for the sand tiger shark *Carcharias taurus* as 120,000% instead of 1,200,000% as originally calculated by Stribling *et al.* [30] and confirmed here (Table 4). Similar transcribing errors can be observed between papers by the same author. The inorganic matter for uterine eggs of the common smoothhound *Mustelus mustelus* was recorded by Ranzi [14] as 0.069 g. However in Ranzi [26], it was recorded as 0.690 g with Needham [20] then recording the value as 0.7 g. Moreover, the value for inorganic matter recorded for uterine eggs of the starry smooth-hound *Mustelus asterias*, changes from 0.050 g [14] to 0.550 g [26]. Needham [20] once again recorded the latter of the two values, 0.550 g, based on Ranzi [26] (Table 4). Transcribing errors, such as these (applying to inorganic matter) can have large ramifications when recalculating organic mass. The implications of this can be observed when looking at the calculations for *Squalus blainvillei* by Ranzi [26] and consequently Needham [20] (Table S1). The organic mass of the egg (10.688 g) and embryo (10.790 g) of *S. blainvillei* were recalculated in this study to be 10.680 g and 9.890 g, respectively, by taking the recorded inorganic matter from the recorded dry mass. Based on this, the organic mass change was calculated here to be −7.397% instead of 1.030% as reported originally by Ranzi [26].

#### Mis-citations

Typographical and rounding errors were amplified when authors summarised previous literature without checking the original values and calculations. For example, the most recent summary of mass change values presented in Hamlett *et al.* [1] is based on Needham [20] and included all the aforementioned mistakes, including the reporting of *Dalatias licha* with a percentage change in inorganic matter of 30% instead of 300%. Furthermore, Hamlett *et al.* [1] reported a dry mass loss from Mellinger *et al.* [17] as −16.8%, for *Scyliorhinus canicula*. However, Mellinger *et al.* [17] provided an organic mass loss of −20.56% or a Carbon plus Hydrogen loss of −26%. In addition, interchanging between dry and organic mass adds to the uncertainty. For example, Wourms *et al.* [11] summarised the changes in organic mass listed in Needham [20] without specifying whether the value referred to organic or dry mass. However in the text, Wourms *et al.* [11] stated that values in the table referred to dry mass.

Taking into consideration the above errors, the most accurate mass change values are provided in Table 5. Species for which lecithotrophy are described, have an organic mass change between −40% and +1%. Placental species appear to have an increase in organic mass greater than or equal to 1000% (Table 5).

**Table 5:** Summary of shark species reproductive mode and percentage change values believed to be the most reliable.

				Percentage Change										
				Wet		Dry		Water Content		Inorganic			Organic	
Species	Study Name	Reproductive mode	Reference	Small^st^	Larg^st^	Small^st^	Larg^st^	Small^st^	Larg^st^	Small^st^	Larg^st^		Small^st^	Larg^st^
*Squatina* *squatina*		Lecithotrophic viviparous	Capapé *et al.* 1990	2.71	−5.09	−48.64	−52.55						CBD ∼0.5	
*Squatina* *oculata*		Lecithotrophic viviparous	Capapé *et al.* 1990	1.78	1.68	−49.11	−49.16						CBD ∼0.5	
*Oxynotus* *centrina*		Lecithotrophic viviparous	Capapé *et al.* 1999										CBD = 1.36	
*Heterodontus* *portusjacksoni ?*		Oviparous	Frazer (unpublished)	57.73	55.25	−33.33	−34.85	263.80	112.07	42.86	20.83		−41.03	−39.81
*Squalas* *acanthias*	*Acanthias* *vulgaris*	Lecithotrophic viviparous	Wourms *et al.* 1988	78.26		−31.82		146.48		300			−39.54	
*Orectolobus* *ornatus ?*		Undefined	Huveneers *et al.* 2011	44	89			169	103	91	56		−32	−33
*Centroscymnus* *coelolepis ?*		Lecithotrophic viviparous	Moura *et al.* 2011	58.95	32.40	−20.98	−29.71	66.01	122.48	29.37	46.49		−22.2	−31.67
*Orectolobus* *maculatus ?*		Undefined	Huveneers *et al.* 2011	45	62			226	120	100	72		−26	−26
*Centrophorus* *granulosus ?*		Lecithotrophic viviparous	Guallart and Vicent 2001	30.59	34.45	−22.80	−14.39	101.30	99.18	114.29	167.57		−25.19	−17.63
*Squalas* *megalops ?*		Lecithotrophic viviparous	Braccini *et al.* 2006	46	58			137	154	100	156		−23	−17
*Dalatias* *licha*	*Scymnus* *lichia*	Aplacental Yolk- sac viviparous	Ranzi 1932	44.62		−12.31		101.54		300.00			−22.22	
*Scyliorhinus* *canicula*	*Scyllium* *canicula*	Oviparous	Ranzi 1932	104.72		−14.20		213.25		292.31			−20.68	
*Chlamydoselachus* *anguineus*		Matrotrophic to some degree	Tanaka *et al.* 1990^a^			7.28								
*Squalas* *blainvillei*	*Acanthias blainvillei*	Lecithotrophic viviparous	Ranzi 1932; 1936	101.61		15.14		214.35		700.00			1.03	
*Galeorhinus* *galeus*	*Geleus* *canis*	Viviparous – unspecified	Ranzi 1936	117.68		26.23		208.94		711.11			10.87	
*Mustelus* *antarcticus*		Minimal histotrophic	Storrie *et al.* 2009; Ranzi 1934	1598.42		684.88		1305.66		1027.27			112.39	
*Mustelus* *asterias*	*Mustelus* *vulgaris*	Aplacental Yolk- sac viviparous	Ranzi 1932	1430.79		416.42		2492.71		2800.00			355.61	
*Mustelus* *mustelus*	*Mustelus* *laevis*	Placental	Ranzi 1932	3325.47		1219.44		5609.98		7610.15			1063.29	
*Carcharias* *glaucus*	*Prionace* *glauca*	Yolk-sac placental	Wourms 1993			2470.00								
*Carcharias* *taurus*	*Eugomphodus* *Taurus*	Intrauterine cannibalism	Stribling *et al.* 1980			1177814.11								
*Pseudotriakis* *microdon*		Aplacental viviparous	Yano 1992	1519900	379900	506567	126567							
*Scoliodon* *laticaudus*		Placental	Wourms 1993			5833844.95								

Based on recalculations made in this study.

?  =  internal yolk sac excluded.

CBD  =  Chemical balance of development.

a =  Tanaka S, Shiobara Y, Hioki S, Abe H, Nishi G, Yano K, Suzuki K (1990) The reproductive biology of the frilled shark, *Chlamydoselachus anguineus*. Jpn J Ichthyol 37:273–291.

## Discussion

The embryonic development of *Heterodontus portusjacksoni* was studied with the aim to determine the validity of using the current threshold value to differentiate aplacental reproductive modes. The results of this study highlight issues with the current threshold value, as the decrease of organic mass from egg to near full-term embryo is vastly different from those recorded for *Scyliorhinus canicula,* the only other oviparous species for which these values have been calculated.

An increase in wet mass (57.73–55.25%) and water content (263.80–112.07%) was observed for *Heterodontus portusjacksoni* from freshly deposited eggs to near full-term embryos. These values are similar or slightly higher than those obtained from other lecithotrophic or incipient histotrophic species studied using the same method (Table 5) [12], [13], [15], [16]. However, when comparing the wet mass gain of *H. portusjacksoni* to the only other oviparous species studied, *Scyliorhinus canicula* (104.72%), it is considerably lower. Yet the percentage change in water content for *S. canicula* (213.25%) lies within the range obtained for *H. portusjacksoni.* The wet mass and water content values obtained in the present study are potentially biased and slightly overestimated due to the burst yolk sac and mixing with additional seawater in some of the freshly deposited egg cases. An attempt was made to account for such bias by conducting a linear regression between the yolk sac wet mass and the amount of mixed seawater included. However, it is possible that the wet mass values and water content values for the freshly deposited eggs were not as accurate as in other studies. This could potentially explain why Figure 2D has the upper limit boundary higher from the scatter points representing near full-term embryos than Figure 2A, 2B or 2C.

The increase in wet mass of the embryonic system is mostly due to the incorporation of water for the development of embryonic tissues. In addition, the slight increase in inorganic mass, related to the formation and calcification of dermal denticles, teeth and vertebrae adds to the overall mass of the system [15]. The increase in inorganic mass (20.83%–42.86%) found in this present study is considerably lower than in other species, especially when compared to *Scyliorhinus canicula* which has an inorganic percentage increase of 292.31%. This may be attributed to the ontogenetic changes observed in *Heterodontus portusjacksoni* dentition [32]. There is a clear progression from juveniles to adults with increasing numbers of teeth rows, teeth in each row, molariform teeth number and size [32]. In addition the molariform teeth of a similar species, with the same feeding method, (the horn shark *Heterodontus francisci*) have been found to be poorly mineralised in juveniles [32], [33].This could be found for *H. portusjacksoni* and would explain the smaller increase of inorganic mass.

There are inherent costs to embryonic development through the yolk transformation process. As a result, unless additional nutrients are added from maternal sources (matrotrophy), the organic mass of an embryonic system is expected to decrease throughout development. This is mainly due to the energy expenditure for growth, standard metabolic requirements and nitrogen excretion [14], [34]. Consequently, the gain or loss of organic mass throughout embryonic development is currently used to determine whether a species is matrotrophic. Based on previous studies the organic mass of an oviparous, and therefore lecithotrophic, species is expected to decrease throughout development by values of ∼20% [14] and 25–30% [11]. Using the loss of dry and organic mass during the embryonic development of an oviparous species, *Scyliorhinus canicula*, and lecithotrophic viviparous species, the gulper shark *Centrophorus granulosus,* Hamlett *et al.* [1] attempted to provide a threshold value of −20% to separate lecithotrophy from matrotrophy. A decrease of less than 20% infers the existence of additional nutrients from maternal sources [1]. However, findings from a recent study showed flaws with this method and suggested that the proposed threshold to determine matrotrophy might not be suitable due to the minimal or possibly inappreciable difference between lecithotrophic species and incipient histotrophic species [13]. Furthermore, confusion lies with this value, as Hamlett *et al.* [1] did not make it clear whether the threshold referred to dry mass or organic mass. When concluding that a loss of 20% is appropriate for oviparous and lecithotrophic viviparous species, values were taken from previous literature pertaining to both dry and organic mass. This has obvious implications for future reproductive classification if they were to be based on this threshold value. For example, Paiva *et al.* [23] referred to the threshold value as a 20% loss in dry mass, however, while Hamlett *et al.,* [1] did not state it clearly, the threshold value actually refers to organic mass (pers. comms. by M. Ellis, co-author of [1] as M. Storrie). When addressing changes in mass, scientists need to label all mass values (wet, dry, water, inorganic and organic) clearly.

This present study is the first to assess changes in mass throughout embryonic development of an oviparous species other than *Scyliorhinus canicula.* However, a direct comparison between the percentage changes of organic values of these two oviparous species should be undertaken with caution because of three main issues.

1. The loss in organic mass observed in *Scyliorhinus canicula* was based on small sample sizes and reported as mean values rather than taking into account biological heterogeneity of the initial egg size. Previous studies have shown that the estimated changes in mass can be biased by small sample size and the assumption of homogeneous egg and embryo sizes [13], [15]. A recent study showed that 50% of mean values obtained for the banded wobbegong *Orectolobus ornatus* and the spotted wobbegong *Orectolobus maculates* did not lie within the range calculated from the theoretical TL-body mass relationship using the method of Guallart and Vicent [15] [13]. Similarly, the mean mass change values calculated for *Heterodontus portusjacksoni* were outside the percentage mass change range calculated for all masses. The mean decrease in organic mass (6%) was ∼6x smaller than the decrease in organic mass calculated when taking into account the biological heterogeneity of initial egg size (41.03–39.81%). Consequently, the mean values reported for *S. canicula* might not be reliable and if another study was conducted on *S. canicula* using the same methods as this present study, the organic decrease could be found to be closer to the values obtained from *H. portusjacksoni*.
2. While several studies investigated the change in embryonic mass throughout development of *Scyliorhinus canicula*, the values were not all reported as organic mass. Very few studies have appropriately dried samples and subsequently incinerated them so that water content and organic and inorganic matter can be accounted for separately [1].
3. Studies on *Scyliorhinus canicula* lacked methodological consistency with the inclusion or exclusion of the IYS. From the methods of more recent studies, it can be established whether or not the IYS was included in the total embryonic mass. However, earlier studies failed to specify this, possibly preventing accurate results and comparisons. In addition, it should be noted that consistency within the methodology was also lacking when regarding the mass of the initial stages of development. Multiple studies used oocytes or ova rather than uterine/fertilised eggs. This leads to an overestimation of percentage change due to the difference in size and composition of oocytes and ova compared with uterine/fertilised eggs [1].

The combination of these issues question the reliability of the values obtained for *Scyliorhinus canicula*. Subsequently, further investigation was carried out on the wider literature to assess the extent of the aforementioned issues. Multiple typographical, calculation and rounding errors and incorrect citations across many papers were revealed (Table S1). Some of these errors resulted in only a minimal influence, however a reporting error of the Mellinger *et al.* [17] value cited by Hamlett *et al.* [1] has large ramifications as the two different values are on either side of the −20% threshold value. If the threshold value was abided by to infer matrotrophy, the reproductive mode of this species (*S. canicula)* would change depending on the paper referred to. Assessments of citation validity have shown that one in four assertions in the ecology and marine biology field are potentially unsubstantiated [35], [36]. It is critical when summarising others work to check the original citation, making sure that what you present is accurate. These discrepancies point out that studies on changes in mass, on which the threshold value is based, can be very inconsistent and possibly unreliable. Therefore suggesting that the current threshold value of −20% is unsound The organic loss of *Heterodontus portusjacksoni* presented in this study is more likely to be accurate and comparable with future studies than *S. canicula.* However, additional factors need to be acknowledged when assessing whether a threshold value can be used to categorise chondrichthyan species within a reproductive mode.

Inter- and intra-species variation is an important consideration when proposing a threshold value While there are no other oviparous species for which the change in organic mass throughout development has been calculated reliably, there are two viviparous species (the piked spurdog *Squalus megalops* and *Centroscymnus coelolepis*) for which lecithotrophy has been confirmed through histological analysis [12], [16]. Since the loss of organic mass was calculated in these two studies using the same methods as the present study, the level of inter-species variation within lecithotrophic species can be better assessed by comparing *Heterodontus. portusjacksoni* to the values obtained from these two species. While the loss of organic mass from freshly deposited eggs to near full-term embryo was ∼40% in *H. portusjacksoni*, it was calculated as ∼27% in *C. coelolepis*, and ∼20% in *S. megalops* [12], [16]. This suggests that a wide inter-species variation does exist, which is not surprising as there is little reason to assume that conversion efficiency from yolk to embryo growth is consistent across taxa, reproductive mode or even across individuals [37]. The variation in yolk conversion efficiency can be attributed to factors such as the physiological state of the female during vitellogenesis [34]. In addition, it has been demonstrated in reptiles and birds that gestation length can play a part in the variation of organic loss between species. Longer gestation periods require metabolic energy expenses for a longer period, resulting in a higher total expenditure (i.e. lesser efficiency of yolk conversion or higher developmental costs overall) [38]. Such processes further make it clear that even with consistent methodology, variables will continue to exist throughout these studies suggesting that separating reproductive modes with minimal difference on a single threshold value, is inherently unsound. Further studies on different oviparous species using the same method as the present study would better determine the level of inter-species variation. In addition, it is likely that *H. portusjacksoni* is distributed as separate populations throughout their range, with the east coast population distinct from those in southern and western Australia [39]. Consequently, a study investigating the change in mass throughout embryonic development of the eastern population of *H. portusjacksoni* would provide additional information about the level of intra-species variability and the potential impacts of environmental conditions.

Considering the previous use of the 20% loss of organic mass to aid the partition of lecithotrophy and matrotrophy, and finding that oviparous species can have an organic mass loss of greater than 20%, it is possible that species previously categorised as lecithotrophic might receive minimal nutrients additional to the EYS and actually exhibit incipient histotrophy. Both *Squalus blainvillei* and *Centrophorus granulosus* have been described as lecithotrophic species based on mass changes, but had an organic loss of less than −40% (Table 5). In addition, a series of papers by Capapé and colleagues [27]–[29] revealed CBD values of ∼0.5, 0.73, and 1.36 for the angel shark species studied, confirming that they are purely lecithotrophic, as the values are described as relatively low [27]–[29]. However, it has been suggested that any CBD value >1 implies matrotrophy [1]. Confusion such as this contributes to the incorrect classification of reproductive modes. It is essential that chondrichthyans are classified accurately to resolve their perplexing reproductive history. Understanding their phylogenetic positioning and evolutionary distinctiveness is essential for conservation planning [40], [41], [42]. In addition, the maternal-embryonic nutritional relationship of species has been found to give great insight into the species risk of extinction when exposed to fishing pressure [43]. Oviparous species are considered to have the highest resilience, decreasing for lecithotrophic viviparous species with adelphotrophy, ovatrophy, histotrophy and placental viviparity having the lowest resilience [43]. Therefore, accurate classification can help infer the resilience to fishing pressure and provide advice on the conservation measures of chondrichthyan species.

### Final Recommendations

The newly obtained values for *Heterodontus portusjacksoni* of −41.03% and −39.81% have important consequences on the previously proposed threshold value. This, supported by the unreliability of previous literature, suggests that the current threshold value of 20% loss in organic mass is unsuitable for discerning between lecithotrophic and incipient histotrophic species. Changes in mass are indicative of general trends and should only be used to separate highly matrotrophic species from lecithotrophic species, as the magnitude of change in organic mass is so large. However, a threshold value, no matter the value, will never be precise enough to solely discern between the specific reproductive modes due to species variation. Reliable changes in mass need to be used in conjunction additional histological methods to allow for the determination of any potential maternal input, and therefore assist in distinguishing between the different reproductive modes.

Currently, the most reliable mass change values are acquired using the methods of Guallart and Vicent [15]. This method can appear quite subjective, however, considering that the two parallel lines representing the extreme variability of freshly deposited egg and full-term embryo masses are drawn arbitrarily. It would be advantageous to develop a function to estimate the placement of these lines to improve credibility.

According to this study and to ensure that comparable values are obtained across future studies, the following are recommended when using mass changes to elucidate the reproductive mode of chondrichthyans:

- Use uterine/fertilised eggs instead of ovarian eggs as the initial masses of the embryonic system;
- Separate the IYS and EYS from the embryo, leaving the eviscerated embryo mass for determining the theoretical TL-embryo mass relationship.
- Use the same incinerating methods as in the present study: obtain dry mass by leaving samples in an oven at 60°C until constant mass is reached, incinerate in a muffle furnace at increasing temperatures to avoid overspill, leaving for two hours at each intermediate temperature (200°C, 300°C and 400°C), and for 15 hrs at a final temperature of 550°C;
- Ensure that each mass (wet, dry, water, inorganic, and organic) are explicitly labelled;
- Use the Guallart and Vicent ponderal methods to account for biological heterogeneity;
- Use the following mass change formula: ((embryo mass-egg mass)/egg mass)*100; and
- Do not rely on a threshold value to discern between lecithotrophic and incipient matrotrophic species, but combine mass change studies with biochemical analysis of uterine fluids and detailed examinations of the micro- and ultrastructure of the uterus.

## Supporting Information

Table S1 **Summary of weight change values reported in previous studies.** All recalculated values are in bold. Percentage weight changes are of the initial value. Species with only one row of values represent the mean weights. Species with two rows of values represent the smallest and largest extremes of variability.
(XLS)

## References

[1] Hamlett WC, Kormanik G, Storrie M, Stevens B, Walker TI (2005) Chondrichthyan Parity, Lecithotrophy and Matrotrophy. In: Hamlett WC (eds) Reproductive Biology and Phylogeny of Chondrichthyes: Sharks, Batoids and Chimaeras. USA: Science Publishers Inc., Enfield, New Hampshire. pp 301–335.

[2] Hamlett WC, Koob TJ (1999) Female reproductive system. In: Hamlett WC (eds) Sharks, Skates and Rays: The Biology of Elasmobranch Fishes. Johns Hopkins University Press, Baltimore, Maryland. pp 339–443.

[3] Hamlett WC (2001) Reproduction in Fish. Encyclopaedia of Life Sciences, John Wiley & Sons, Ltd. pp 1–7.

[4] Hisaw FL, Albert A (1947) Observations on the reproduction of the spiny dogfish, *Squalus acanthias.* . Biol Bull 92: 187–199. 20249523

[5] Wourms JP (1977) Reproduction and development in Chondrichthyan fishes. Am Zool 17: 379–410.

[6] Gilbert PW, Schlemitzauer DA (1966) The placenta and gravid uterus of *Carcharhinus falciformis.* . Copeia 1996: 451–457.

[7] Hamlett WC, Wourms JP, Hudson JS (1985) Ultrastructure of the full-term shark yolk sac placenta I. Morphology and cellular transport at the fetal attachment site. J Ultrastruct Res 91: 192–206. 4094013 10.1016/s0022-5320(85)80013-7

[8] Otake T, Mizue K (1985) The fine structure of the placenta of the blue shark *Prionace glauca* . Jpn J Ichthyol 32: 52–59.

[9] Hamlett WC, Sever DM, Hysell CK (1999) Gestational plasticity of the uterus in placental sharks. Placenta 20: A28.

[10] Wourms JP (1981) Viviparity: the maternal-fetal relationship in fishes. Am Zool 21: 473–515.

[11] Wourms JP, Grove BD, Lombardi J (1988) The maternal-embryonic relationship in viviparous fishes. In: Hoar WS, Randall DJ (eds) Fish Physiology. San Diego: Academic Press. 1–134.

[12] Moura T, Nunes C, Bandarra N, Gordo LS, Figueiredo I (2011) Embryonic development and maternal–embryo relationships of the Portuguese dogfish *Centroscymnus coelolepis.* . Mar Biol 158: 401–412.

[13] Huveneers C, Otway N, Harcourt R, Ellis M (2011) Quantification of the maternal-embryo nutritional relationship of elasmobranchs: case study of wobbegong sharks (genus *Orectolobus*). J Fish Biol 78(5): 1375–1389. 21539548 10.1111/j.1095-8649.2011.02938.x

[14] Ranzi S (1932) Le basi fisio-morfologiche dello sviluppo embrionae dei Selaci. Parte I. PSZNI 13: 209–290.

[15] Guallart J, Vicent J (2001) Changes in composition during embryo development of the gulper shark, *Centrophorus granulosus* (Elasmobranchii, Centrophoridae): an assessment of maternal-embryonic nutritional relationships. Environ Biol Fish 61: 13–150.

[16] Braccini JM, Hamlett WC, Gillanders BM, Walker TI (2007) Embryo development and maternal-embryo nutritional relationships of piked spurdog (*Squalus megalops*). Mar Biol 150: 727–737.

[17] Mellinger J, Wriez F, Alluchon-Gerard MJ (1986) Developmental biology of an oviparous shark, *Scyliorhinus canicula.* In: Uyeno T, Arai R, Taniuchi T, Matsuura K (eds) Indo-Pacific Fish Biology. Proc 2nd International Conference on Indo-Pacific Fishes Tokyo: Ichthyological Society of Japan, Tokyo. 310–332.

[18] Delhaye E, Lechenault H, Wrisez F, Leray C, Haye B, et al (1992) Localisation, composition et utilisation des lipides vitellins chez *Scyliorhinus canicula.* . Bull Soc Zool Fr 117: 149–156.

[19] Lechenault H, Wrisez F, Mellinger J (1993) Yolk utilization in *Scyliorhinus canicula,* an oviparous dogfish. Environ Biol Fish 38: 241–252.

[20] Needham J (1942) Biochemistry and Morphogenesis, London: Cambridge University Press.

[21] Rodda K, Seymour R (2007) Functional morphology of embryonic development in the Port Jackson shark *Heterodontus portusjacksoni* (Meyer). J Fish Biol 72: 961–984.

[22] Storrie M (2009) Microscopic modifications of the reproductive tissues of the gummy shark (*Mustelus antarcticus*) during maturation and gestation. Ph.D. dissertation, Deakin University, Victoria, Australia, 153 p.

[23] Paiva RB, Neves A, Sequeira V, Nunes ML, Gordo LS, et al (2012) Reproductive strategy of the female deep-water shark birdbeak dogfish, *Deania calcea:* lecithotrophy or matrotrophy? J Mar Biol Assoc U. K. 92(2): 387–394.

[24] Wourms JP (1993) Maximization of evolutionary trends for placental viviparity in the spadenose shark, *Scoliodon laticaudus.* . Environ Biol Fish 38: 269–294.

[25] Ranzi S (1934) Le basi fisio-morfologiche dello sviluppo embrionae dei Selaci. Parte II e III. PSZNI 13: 332–437.

[26] Ranzi S (1936) Reproduction in selachians. Naturwissenschaften 24: 642–646.

[27] Capapé C, Quignard JP, Mellinger J (1990) Reproduction and development of two angel sharks, *Squatina squatina* and *S. oculata* (Pisces: Squatinidae), off Tunisian coasts: semi-delayed vitellogenesis, lack of egg capsules, and lecithotrophy. J Fish Biol 37: 347–56.

[28] Capapé C, Seck AA, Quignard JP (1999) Observations on the reproductive biology of the angular rough shark, *Oxynotus centrina* (Oxynotidae). Cybium 23: 259–271.

[29] Capapé C, Secko AA, Gueye-Ndiaye A, Diatta Y, Diop M (2002) Reproductive biology of the smoothback angel shark, *Squatina oculata* (Elasmobranchii: Squatinidae), from the coast of Senegal (eastern tropical Atlantic). J Mar Biol 82: 635–640.

[30] Stribling MC, Hamlett WC, Wourms JP (1980) Developmental efficiency of oophagy, a method of viviparous embryonic nutrition displayed by the Sand Tiger Shark (*Eugomphodus laurus*). Proc North Carolina Acad Sci 42: 111.

[31] Yano K (1992) Comments on the reproductive mode of the false cat shark *Pseudotriakis microdon* . Copeia 21: 460–468.

[32] Powter DM, Gladstone W, Platell M (2010) The influence of sex and maturity on the diet, mouth morphology and dentition of the Port Jackson shark, *Heterodontus portusjacksoni.* . Mar Freshw Res 61: 74–85.

[33] Summers AP, Ketcham RA, Rowe T (2004) Structure and function of the horn shark (*Heterodontus francisci*) cranium through ontogeny: development of a hard prey specialist. J Morphol 260: 1–12. 15052592 10.1002/jmor.10141

[34] Heming TA, Buddington RK (1988) Yolk absorption in embryonic and larval fishes. In: Hoar WS, Randall DJ (eds) Fish Physiology. San Diego: Academic Press. 407–466.

[35] Todd PA, Yeo DC, Li D, Ladle RJ (2007) Citing practices in ecology: can we trust our own words? Oikos 116: 1599–1601.

[36] Todd PA, Guest JR, Lu J, Ming Chou L (2010) One in four citations in marine biology papers is inappropriate. Mar Ecol Prog Ser 408: 299–303.

[37] Blackburn DG (1994) Standardised criteria for the recognition of embryonic nutritional patterns in squamate reptiles. Copeia 1994: 925–935.

[38] Birchard GF, Walsh T, Roscoe R, Reiber CL (1995) Oxygen uptake by Komodo dragon (*Varanus komodoensis*) eggs: the energetics of prolonged development in reptiles. Physiol Zool 68: 622–633.

[39] O'Gower AK, Nash AR (1978) Dispersion of the Port Jackson shark in Australian waters. In: Hodgson ES, Mathewson RF (eds) Sensory Biology of Sharks, Skates, and Rays. Arlington, VA: Office of Naval Research, Department of the Navy. pp 529–544.

[40] Steel M, Mimoto A, Mooers AØ (2007) Hedging one's bets: quantifying a taxon's expected contribution to future phylogenetic diversity. Evol Bioinform 3: 237–244. PMC2684137 19461983

[41] Isaac NJB, Turvey ST, Collen B, Waterman C, Baillie JEM (2007) Mammals on the edge: conservation priorities based on threat and phylogeny. PLoS ONE 2(3): e296. 17375184 10.1371/journal.pone.0000296 PMC1808424

[42] Mooers AØ, Faith DP, Maddison WP (2008) Converting endangered species categories to probabilities of extinction for phylogenetic conservation prioritization. PLoS ONE 3(11): e3700. 19002251 10.1371/journal.pone.0003700 PMC2577618

[43] Garcia VB, Lucifora LO, Myers RA (2008) The importance of habitat and life history to extinction risk in sharks, skates, rays and chimaeras. Proc R Soc 275: 83–89. 10.1098/rspb.2007.1295 PMC2562409 17956843

